# Sex-dependent effects of the targeted nerve growth factor mutation (R100E) on pain behavior, joint inflammation, and bone erosion in mice

**DOI:** 10.1097/j.pain.0000000000003343

**Published:** 2024-09-25

**Authors:** Carlos E. Morado-Urbina, Jungo Kato, Katalin Sandor, Juan Antonio Vazquez-Mora, Kristina Ängeby Möller, Nils Simon, Jaira Salcido, Arisai Martinez-Martinez, Enriqueta Munoz-Islas, Juan Miguel Jimenez-Andrade, Camilla I. Svensson

**Affiliations:** a Department of Physiology and Pharmacology, Center for Molecular Medicine, Karolinska Institutet, Solna, Sweden; b Department of Anesthesiology, Keio University School of Medicine, Tokyo, Japan; c Unidad Académica Multidisciplinaria Reynosa Aztlán, Universidad Autónoma de Tamaulipas, Reynosa, México

**Keywords:** Nerve growth factor, Joint swelling, Joint pain insensitivity, Sex-dependent mechanisms, Bone loss

## Abstract

Nerve growth factor (NGF)-R100E is a mutated form of human recombinant NGF that reduces the binding of NGF to its p75NTR receptor while retaining its affinity toward the TrkA receptor. Here, we used human wild type NGF and NGF-R100E knock-in mice to investigate the effects of this NGF mutation on inflammation-induced pain-related behaviors and bone loss. The hNGF-R100E mutation did not alter the nerve fiber density in the sciatic nerve, ankle joint synovium, and skin of naïve mice. Withdrawal responses to mechanical, thermal, and cold stimuli before and after joint inflammation induced by intra-articular injection of complete Freund adjuvant (CFA) were similar between human recombinant nerve growth factor-wild type and hNGF-R100E male and female mice while weight bearing and gait analysis revealed significant differences. Intriguingly, hNGF-R100E male and female mice showed only mild changes, indicating lower degrees of deep joint–related pain compared to their wild type counterparts. Furthermore, micro-CT analysis demonstrated that hNGF-R100E female mice, but not males, were protected from CFA-induced bone loss, and mRNA analysis showed a different gene regulation indicating a sex-dependent relationship between NGF, inflammation, and bone loss. In conclusion, our study reveals that the hNGF-R100E mutation renders mice insensitive to inflammation-induced impact on joint loading and gait while preserving the development of the peripheral nociceptive neurons and sensitivity to punctate stimulation of the skin. Notably, the mutation uncovers a sex-dependent relationship between NGF and inflammation-induced bone loss. These findings offer valuable insights into NGF as a target for pain management and the interplay between NGF and bone architecture.

## Abstract 2

hNGF-R100E mutation in mice protects against complete Freund adjuvant–induced joint pain in both sexes and bone loss in females.

Supplemental Digital Content is Available in the Text.

## 1. Introduction

Nerve growth factor (NGF) is one candidate tested as a potential therapeutic target for chronic pain. Numerous preclinical and clinical studies have shown local and systemic upregulation of NGF in chronic pain conditions such as rheumatoid arthritis, osteoarthritis, cancer, and degenerative intervertebral disc disease,^1,4,18,19,47,48^ suggesting an active role of NGF in pain. Consistently, anti-NGF treatment is effective in reducing mechanical and thermal hypersensitivity in chronic pain models in rodents.^23,26,49^ Based on these findings, several clinical trials testing the effectiveness of anti-NGF antibodies in chronic pain were conducted and significant pain relief was achieved for osteoarthritis, low back, visceral, and cancer pain.^10,28,30,40,50^ Importantly, the risk of adverse events potentially associated with sequestering NGF, such as peripheral sensory abnormalities, osteonecrosis, and arthropathy in joints with osteoarthritis, have also been pointed out, eventually leading to the suspension of these trials.^29^ Therefore, it is crucial to identify more specific NGF signaling involved in the pathogenesis of chronic pain and develop a therapeutic approach that selectively blocks pain-specific NGF signaling pathways.

The genetic analyses of northern Swedish individuals with a rare pain-free congenital disorder called hereditary sensory and autonomic neuropathy type V (HSAN V)^6^ revealed a mutation in the *ngf* gene in which arginine 100 was replaced by tryptophan (R100W), attracting attention as a disease that encompasses the specific role of NGF in pain. Hereditary sensory and autonomic neuropathy type V is unique because it selectively affects deep pain perception while cognition, sensory, and autonomic functions remain relatively preserved.^21,37^ Because of the insensitivity to deep pain and the lack of protective reflexes, HSAN V patients often suffer from severe orthopedic problems such as joint inflammation, destruction, and fracture.^36^

A further understanding of the pathogenesis underlying HSAN V has been hampered by the early lethality of the homozygous transgenic mice carrying the human NGF-R100W (hNGF-R100W) mutation. To circumvent this problem, we have used a knock-in mouse expressing mutant human NGF in which arginine is substituted by glutamic acid in the residue R100 in mature NGF (R100E). The in vitro characterization of the hNGF-R100E mutation demonstrates that, similarly to R100W, it yields NGF with a high affinity to the TrkA receptor and a reduced binding capacity to p75NTR. However, in contrast to hNGF-R100W, the maturation and secretion of hNGF-R100E are not altered in vitro.^11^ In this study, we characterized the in vivo effects of the hNGF-R100E mutation on pain-related behaviors and bone density. Our work revealed that hNGF-R100E mice exhibit signs of insensitivity to complete Freund adjuvant (CFA)-induced changes in behavior that is dependent on joint loading, such as weight bearing and gait parameters, while the development of the nociceptive nervous system and sensitivity to mechanical and thermal stimulation of the skin remain preserved. Interestingly, the hNGF-R100E mutation prevents inflammation-induced bone loss in female but not male mice. These data provide novel evidence that even minimal alterations in NGF caused by the hNGF-R100E mutation are sufficient to induce insensitivity to reduce nociceptive signaling in vivo and that there may be a sex-related dimorphism linked to the role of NGF in bone metabolism.

## 2. Methods

### 2.1. Animals

The local ethical committee approved all experiments for animal experiments in Sweden (Stockholms Norra Djurförsöksetiska Nämnd; N622/12, N108/13, N151/16, and 12127-2020).

Human wild type (WT) NGF and human NGF-R100E knock-in mice on C57BL/6 background were a kind gift from AstraZeneca, Södertälje, Sweden. Briefly, the mice were produced by using a targeting construct containing human NGF with or without the R100E mutation transfected into embryonic stem cells (ESCs) by electroporation (Fig. S1, http://links.lww.com/PAIN/C98). Gene-targeted homologous recombinant clones were identified by Southern blot analysis of genomic DNA isolated from individual neomycin-resistant ESCs colonies. Thereafter, appropriately targeted ESCs were microinjected into donor blastocysts and then implanted into pseudo-pregnant foster mothers to obtain chimeric mice. Chimeric males were crossed with C57BL/6 females to get heterozygote mice. Heterozygous mice were interbred to obtain homozygous offspring. Mice were genotyped by polymerase chain reaction (PCR) (human NGF insert) and sequencing (mutation). The following PCR primers were used: forward primer 5-GTC​CTT​TCT​CTC​AGT​AAG​AGG​GGG​AAG​GAG​GGA​AGA​CAT​A-3; reverse primer 5-GCC​TCT​ACT​TAT​CCA​CAC​TGG​ATT​CCC​TTA​GGA​AGG​TTC​TGG-3. Band sizes: WT band 390 bp; human NGF band 448 bp. The transgenic animals were bred at the Karolinska Institutet animal facility in a specific pathogen-free room. Mice were housed in standard cages (3-5 animals per cage) under a climate-controlled environment with a 12-hour light/dark cycle. Food and water were provided ad libitum. In this study, adult female and male transgenic mice between 6 and 10 months of age were used. In addition, C57BL/6JRj WT male mice, 12 to 16 weeks of age (Janvier Labs, Le Genest-Saint-Isle, France), were also used for some comparative immunohistochemical studies. All behavioral experiments were performed during the light period.

### 2.2. Behavioral tests

Mice were habituated to the test environment 2 times before baseline testing. On testing days, the mice were placed in acrylic chambers (13 × 13 × 13 cm) on top of a wire mesh grid floor or glass floor and left to habituate. Experimenters were blinded to the transgenic strain of mice during the behavioral assessments.

For mechanical sensitivity, paw withdrawal thresholds in response to von Frey filaments (OptiHair, Marstock nervtest, Schriesheim, Germany) were assessed. A series of filaments with a logarithmically incremental stiffness of 0.5, 1, 2, 4, 8, 16, and 32 mN (converted to 0.051 g, 0.102 g, 0.204 g, 0.408 g, 0.815 g, 1.63 g, and 3.26 g, respectively) were applied perpendicularly to the plantar surface of the hind paw in an up-down fashion starting with the 4 mN filament and held for 3 seconds. A positive response was noted if the paw was withdrawn. The 50% withdrawal threshold was determined using the up-down method, based on Dixon model^20^ as previously described.^15^ The baseline for mechanical sensitivity was assessed on 3 separate days, with one day in between tests, and values were averaged.

Thermal sensitivity was assessed using Hargreaves apparatus.^25^ The mice were habituated to acrylic chambers (13 × 13 × 13 cm) on a glass floor (3 mm thick) maintained at 32°C. A beam of light was directed to the plantar surface of the hind paw, and both the increasing temperature and the latency until paw withdrawal were measured. To prevent tissue damage to the paw, the cutoff was determined as 20 seconds, and the maximum temperature was 50°C. The test was repeated 3 times for each paw, and the mean value was calculated. The average of 3 baseline values was calculated based on recordings obtained on 3 separate occasions 1 to 2 days apart.

Cold sensitivity was assessed using a modification of the acetone drop method^59^ (supplementary methods, http://links.lww.com/PAIN/C98) and a modification of the cold plantar assay.^9^ In the cold plantar assay, the cold stimulus (dry ice pellet) was applied to the hind paw through the glass after habituation to the acrylic chambers (13 × 13 × 13 cm) placed on a glass surface (4 mm thick). The dry ice pellet with a smooth and flat surface (approximately 0.8 cm in diameter) was pressed to the glass under the hind paw and held firmly until the mouse lifted the paw or walked away. Response time was recorded using a manual chronometer. The stimulation time was limited to 15 seconds to prevent cold-induced tissue damage. The baseline was assessed on 3 days, with 2 to 3 days between tests, and the values averaged. To prevent sensitization because of the repeated stimuli in the mice, mechanical, cold, and thermal tests were conducted on different days.

Weight bearing was assessed using the Advanced Dynamic Weight Bearing (DWB) apparatus (Bioseb, Vitrolles, France). Each mouse was acclimatized to the Plexiglas chamber (11 × 11 × 19.7 cm). For baseline and testing, the mice were allowed to move freely for 5 minutes in the Plexiglas chamber with a sensor mat (TecScan, Quebec, Canada) containing pressure transducers while a camera recorded the movement from the top. The percentage of weight borne by the ipsilateral paw over the total body weight and the time spent guarding the ipsilateral paw during the 5-minute observation period were used as parameters in this study. One baseline was recorded 1 to 2 days before testing.

Gait analysis was performed using the CatWalk device (Noldus, Wageningen, The Netherlands). The footprints were captured while the mouse voluntarily traversed a 100-cm long walkway consisting of a glass floor with black acrylic walls placed 10 cm apart. Light was projected into one long edge, and the design allowed for virtually complete reflection of the light within the floor except where an object, such as a mouse paw, touches the glass, causing the light to be scattered and produce an illuminated image.^24^ Training on the equipment was performed 2 to 3 times before the baseline was recorded. During baseline and testing, the mice were allowed to voluntarily cross the walkway until 3 satisfactory crossings were recorded (runs with a minimum duration of 0.5 seconds, maximum duration of 8.0 seconds, and an allowed variation of 65%). In this study, different parameters were used to assess changes in gait. The regularity index reflects the interlimb coordination; the duty cycle measures the proportional duration of the paw placement on the floor during a step cycle; stride length represents the distance between each step of a paw during a step cycle; and the swing speed indicates the paw's velocity while moving to the next step.

### 2.3. Complete Freund adjuvant–induced ankle or knee joint inflammation

Under isoflurane anesthesia, 5 µL or 10 µL of the complete Freund adjuvant (CFA, 10 mg/mL, Chondrex, Woodinville, WA) were injected into the tibiotarsal or knee joint, respectively, using a 29-G needle connected to a 20-μL Hamilton syringe with a plastic cannulae.^16,22^ Injection of saline or the noninjected contralateral side was used as a control. The development of joint swelling was assessed by measuring the ankle or knee joint thickness using a digital caliper. Data are shown as % of swelling increase of the injected joint compared with the noninjected contralateral joint.

### 2.4. Tissue harvest and preparation

Snap-frozen and formalin-fixed tissues were collected. Snap-frozen ankle joints were collected and stored at −70°C. Fixed tissue was collected from isoflurane-anesthetized mice after transcardiac perfusion with 0.01 M phosphate-buffered saline (PBS, Merck, Darmstadt, Germany) followed by 4% formaldehyde (Histolab, Askim, Sweden). The L5 dorsal root ganglion (DRGs), sciatic nerve (approximately 5 mm length above the knee level), glabrous skin, ankle, and knee joints were harvested; maintained in the same fixation solution overnight’ and cryoprotected in 20% sucrose in 0.01 M PBS. The DRGs, sciatic nerve, and glabrous skin were embedded in optimum cutting temperature compound (OCT, Histolab) and serially cut on a cryostat (CryoStar NX70, Thermo Scientific, Stockholm, Sweden) (thickness: skin 30 µm; sciatic nerve and DRG 12 μm), mounted on glass slides, and stored at −20°C until analysis. The ankle and knee joints were maintained in 0.01 M PBS until the microCT analysis and then decalcified using a 0.5 M ethylenediaminetetraacetic acid tetrasodium salt (EDTA, Merck) solution at 4°C for 2 weeks or until total decalcification. The decalcification was monitored by plain X-ray (Fona X70, Fona, Assago, Italy). After decalcification, joints were cryoprotected in 30% sucrose at 4°C for 48 hours and serially sectioned in a cryostat (Leica 1900, Leica Biosystems, Deer Park, IL) along the longitudinal axis at a thickness of 20 μm.

### 2.5. Immunohistochemistry

#### 2.5.1. Dorsal root ganglion and sciatic nerve

The sections were rehydrated with PBS, permeabilized with 0.2% Triton X (X-100, Merck) in PBS, and incubated with 5% normal goat (10000C, ThermoFisher, Stockholm, Sweden) or donkey serum (S30, Merck) in 0.2% Triton X/PBS to block nonspecific binding. The sections were then incubated overnight with the primary antibodies: anti-TrkA (1:100, AF1056, R&D Systems, Minneapolis, MN), anti-CGRP (1:2000, C8198, Sigma, Stockholm, Sweden), and anti-Tuj-1 (1:2000, G7121, Promega, Madison, WI). The immunoreactivity was visualized using the secondary antibodies conjugated to Alexa 488 (1:300, Life Technologies), Alexa 555 (1:300, Life Technologies, Stockholm, Sweden), Cy2 (1:200, Jackson Laboratory, West Grove, PA), or Cy3 (1:600, Jackson Laboratory). Sections were counterstained with 4′,6-diamidino-2-phenylindole, dihydrochloride (DAPI, 1:20000, Invitrogen, Paisley, United Kingdom) and coverslipped with Prolong Gold mounting media (P36934, ThermoFisher).

#### 2.5.2. Skin innervation

Slides were blocked with a 0.01M PBS solution containing 3% normal donkey serum (NDS) and 0.3% Triton X-100. Slides were then incubated with an anti-PGP9.5 (1:1000, CL7756AP, Cedarlene, Stockholm, Sweden), anti-CGRP (1:2000, C8198, Sigma), anti-TrkA (1:50, AF1056, R&D Systems), or an anti-NF200 (1:2000, CH22104, Neuromics, Edina, MN) overnight at room temperature. The next day, an appropriate secondary antibody was used for each primary antibody, then counterstained with DAPI (1:20000, Invitrogen) dehydrated through an alcohol gradient, cleared with xylene, and finally coverslipped with permanent mounting media (Pertex, 00811, Histolab).

#### 2.5.3. Joint innervation

After rehydration with PBS, the tissue sections were incubated with blocking solution for 2 hours. Thereafter, sections were incubated with anti-CGRP (1:2000, C8198, Sigma) and anti-TrkA (1:100, AF1056, R&D Systems) primary antibodies for 12 hours. Then, after washing 3 times with PBS, appropriate secondary antibodies were applied: donkey antirabbit Cy3 (1:600 Jackson ImmunoResearch, West Grove, PA) and donkey antigoat Cy5 (1:500 Jackson ImmunoResearch, West Grove, PA) for 3 hours. The slides were washed with PBS and counterstained with DAPI (1:20000, Sigma Aldrich, St. Louis, MO) dehydrated through an alcohol gradient, cleared with xylene, and finally coverslipped with DPX mounting media (Sigma Aldrich).

#### 2.5.4. Image analysis

The quantification of sciatic nerve fibers labeled with the antibodies described above was performed using a custom script; 20 lines at even intervals throughout the region of interest were drawn perpendicularly to the direction of axons, and the pixel intensities along each line were plotted. The intensity matrices were then processed through a peak-detection algorithm to detect the labeled sciatic nerve fibers represented as positive intensity peaks on individual line plots. The proportion of CGRP-positive fibers in Tuj1-positive sciatic nerve fibers was analyzed in 4 to 5 sections per animal (with a minimum of 30 μm apart), and the average value for each animal was calculated; a total of 4 mWT-NGF, 5 hWT-NGF, and 5 hR100E-NGF mice were included. For quantifying the proportion of TrkA-positive neurons in L5 DRG, the number of TrkA-positive neurons was counted and divided by the number of PGP9.5 (pan-neuronal marker)-positive neurons. Four to 5 sections per animal (with a minimum of 80 μm apart) were analyzed, and the average value for each animal was calculated in 5 mice from each genotype were included. Images for sciatic nerve and DRG analysis were acquired using an inverted fluorescence microscope (Nikon Eclipse TE300, Langen, Germany).

The number of fibers crossing the basal layer was assessed in glabrous skin using the fiber nerve counting rules.^32,39^ Glabrous skin Z-stack images were collected using a Zeiss LSM800 confocal microscope operated by LSM ZEN2012 software (Zeiss, Oberkochen, Germany) and then transformed to a 2-dimensional maximum projection image for analysis. Four to 5 sections per animal (with a minimum of 80 μm apart) were analyzed, and the average of each animal was calculated; a total of 6 hWT-NGF and 6 hR100E-NGF mice were included. Three different confocal images per joint were acquired with a Zeiss LSM800 confocal microscope at 40X magnification. Nerve profiles in the joint were quantified using the ImageJ software (National Institutes of Health), where nerve profiles were manually counted. Four to 11 mice from each sex and genotype were included in the analysis. Data are shown as the density of nerve profiles per volume of the synovium (mm/mm^3^). Experimenters were blinded during image analysis.

### 2.6. Bone analysis

To analyze changes in bone after CFA-induced inflammation, the ankle joint was scanned using a microCT system (SkyScan 1272, Brucker, Kontich, Belgium). The scanning was performed according to the guidelines for microCT analysis for rodent bone structure.^8^ In brief, a 10 µm voxel size, 60 kVp x-ray power, and 166 µA with an integration time of 627 ms at 2016 × 1344 pixel resolution images were acquired and reconstructed using NRecon software (Brucker). To determine the region of interest in the calcaneus bone, the calcaneus was located vertically at 0.5 mm from the beginning of the bone. A 1 mm region was taken, which was delimited by a 0.55 cylinder to take only the trabecular bone. The bone density (BMD), percent bone volume (BV/TV), trabecular number (Tb.N), trabecular separation (Tb.Sp), and trabecular thickness (Tb.Th) were automatically analyzed in the CTAN software (Brucker). Hydroxyapatite calibration dummies (250 and 750 mg/cm^3^) were used to calibrate bone density.

### 2.7. Quantitative real-time polymerase chain reaction

To assess the expression levels of mRNA related to cytokines, osteoclast, macrophages, or neutrophils within the inflamed joints of hNGF-R100E mice, we used ankle joints harvested from both hNGF-R100E mutant mice and human recombinant nerve growth factor-wild type (hNGF-WT) mice on day 8 after intra-articular injection of CFA. In brief, the ankle joints were mechanically disrupted using a BioPulverizer (BioSpec, Bartlesville, OK) and subsequently homogenized using a TissueLyser II (Qiagen, Hilden, Germany) in TRI Reagent (Sigma). We conducted total RNA extraction in accordance with the manufacturer's instructions, and subsequently, reverse transcription was conducted using the high-capacity cDNA reverse transcription kit (Invitrogen). Quantitative PCR was performed using preestablished hydrolysis probes to quantify relative mRNA levels (Table 1). Data were normalized against the housekeeping gene (Rplp2), and relative fold changes were calculated using the comparative Ct method (2-ΔΔCT).

**Table 1:** List of hydrolysis probes used for real-time polymerase chain reaction assay.

Gene	Species	Assay ID	Dye
*Acp5*	Mouse	Mm00475698_m1	FAM-MGB
*Cd163*	Mouse	Mm00474091_m1	FAM-MGB
*Clcn7*	Mouse	Mm00442400_m1	FAM-MGB
*Ctks*	Mouse	Mm00484039_m1	FAM-MGB
*CXCL1*	Mouse	Mm04207460_m1	FAM-MGB
*IL-10*	Mouse	Mm00439616_m1	FAM-MGB
*IL-6*	Mouse	Mm00446190_m1	FAM-MGB
*Itgam*	Mouse	Mm00434455_m1	FAM-MGB
*Mpo*	Mouse	Mm01298424_m1	FAM-MGB
*Rplp2*	Mouse	Mm03059047_gH	FAM-MGB
*Tcirg1*	Mouse	Mm00469406_m1	FAM-MGB
*Tnf*	Mouse	Mm00443258_m1	FAM-MGB

### 2.8. Statistics

All statistical analyses were performed using GraphPad Prism 9. Data are presented as mean values ± SEM. The results obtained from the immunohistology analysis were compared using one-way ANOVA. The baseline sensory comparison and the hypersensitivity to mechanical and thermal stimuli after injection of CFA between hWT and hR100E mice were subjected to Student *t* test, as was the AUC for the formalin test results of the joint swelling. Two-way ANOVA followed by Bonferroni multiple comparisons test vs baseline values was performed for the assessment of weight bearing, guarding, regularity index, and the duty cycle results from more than two time points after i.a. injection of CFA. When only two time points were assessed after CFA injection, Student *t* test was used to test weight bearing and guarding vs baseline values. The quantitative PCR results were compared by an unpaired Mann–Whitney test. A *P*-value of less than 0.05 was considered significant.

## 3. Results

### 3.1. Nociceptive neuron development in mice expressing human WT and R100E nerve growth factor

**Figure 1.**
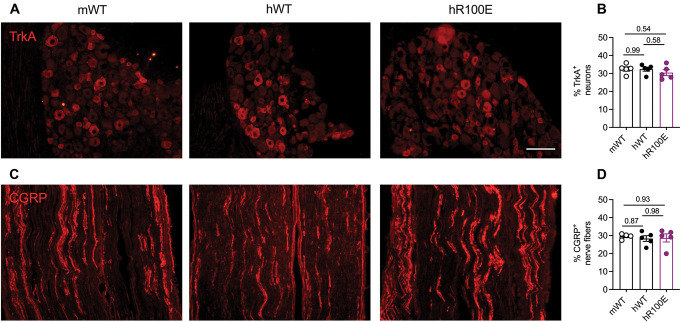
Normal development of TrkA-expressing neurons in the L5 DRG and C peptidergic fibers in the sciatic nerve. Expression of the TrkA receptor (A) in mNGF-WT, hNGF-WT, and hNGF-R100E mouse DRG with no significant differences in the number of TrkA^+^ neurons between the mouse genotypes (B). Expression of CGRP (C) in the sciatic nerve of mNGF-WT, hNGF-WT, and hNGF-R100E mice. No significant differences in the proportion of CGRP fibers were found (D). Data are presented as mean ± SEM. Each dot represents one mouse. n = 4 to 5 mice/group. Scale bar = 100 μm. CGRP, calcitonin gene-related peptide; DRG, dorsal root ganglion; TrkA, tropomyosin receptor kinase A.

**Figure 2.**
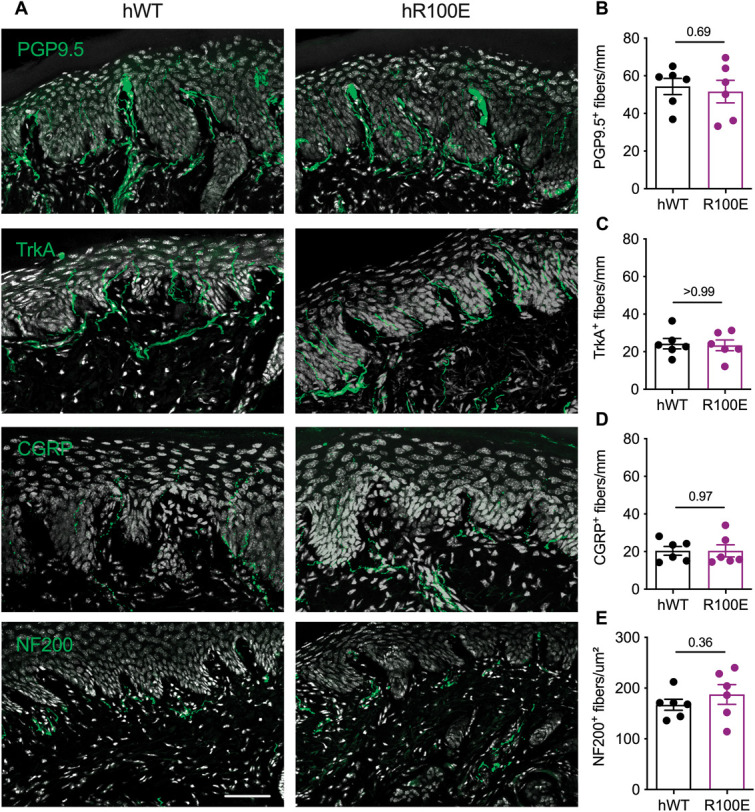
The hNGF-R100E mutation does not alter skin innervation. Representative confocal skin images of hNGF-WT and hNGF-R100E male mice (A) showing PGP9.5, TrkA, CGRP, and NF200 immunoreactivity in green and nuclei in white. No significant differences in the intraepidermal nerve fiber density of PGP9.5 (B), TrkA (C), CGRP (D), or dermal NF200 nerve profile density (E). Each dot represents one mouse, n = 6 mice/group. Data are presented as mean ± SEM. Scale bar = 50 μm. CGRP, calcitonin gene-related peptide; NF200, neurofilament 200; PGP9.5, protein gene product 9.5; TrkA, tropomyosin receptor kinase A.

To examine whether the development of nociceptive neurons is affected by humanization of the NGF gene or introduction of the hNGF-R100E mutation in mice, we compared the proportion of nociceptive neurons in the L5 DRG and sciatic nerve and the density of nerve fibers in synovium and joints in these mice by immunohistochemistry. First, the amount of TrkA-expressing nociceptive neurons in the L5 DRGs from WT mice was similar in magnitude to DRGs from hNGF-WT and hNGF-R100E mice (Fig. 1A, B). Likewise, there was no significant difference in the density of CGRP-positive axons in the sciatic nerves between hNGF-WT, hNGF-R100E, and mNGF-WT mice (Fig. 1C, D). Consistent with the close homology between human and murine NGF genes,^56^ these data suggest that human NGF functions as a neurotrophic factor for developing nociceptive neurons in mice. In addition, we investigated whether hNGF-R100E mice displayed a decrease in nociceptive fibers in the skin (Fig. 2A). We did not find a notable change in the density of PGP9.5^+^ (Fig. 2B), TrkA^+^ (Fig. 2C), CGRP^+^ (Fig. 2D), or NF200^+^ (Fig. 2E) nerve fibers between the hNGF-R100E and the hNGF-WT mice. Thus, taken together, these data suggest a normal development of nociceptor neurons in mice with the hNGF-R100E mutation.

### 3.2. Nociceptive pain-like behavior responses of mice expressing hNGF-R100E

**Figure 3.**
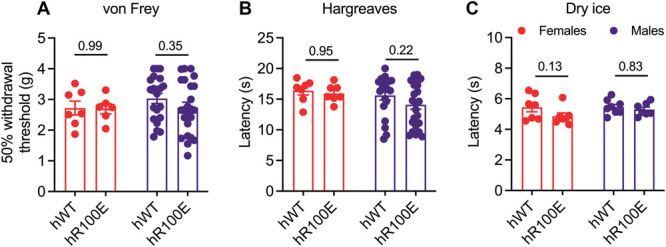
The hR100E mutation does not alter the nociceptive response to mechanical, thermal, or cold stimuli in naïve mice. No significant differences in the withdrawal thresholds to mechanical (von Frey filaments) (A), heat (Hargreaves box) (B), and cold (dry ice assay) (C) stimuli between hNGF-WT and hNGF-R100E naive mice. Data are presented as mean ± SEM. Each dot represents one mouse. For mechanical thresholds, n = 7 females and 20 to 24 males/group; heat thresholds, n = 7 females and 20 to 24 males/group; dry ice test, n = 7 females and 7 to 8 males/group.

While cognition is not altered in heterozygous R100W mice, they display impaired nociception accompanied by decreased skin innervation.^53,58^ To investigate if hNGF-R100E mice exhibit signs of sensory abnormalities, we compared the responsiveness to mechanical, heat, and cold stimuli between naïve hNGF-WT and hNGF-R100E mice. The withdrawal response threshold to mechanical stimulation with von Frey filaments was similar between hR100E and hWT mice (Fig. 3A). Similarly, the latency to heat application was also comparable between hNGF-R100E and hNGF-WT mice (Fig. 3B). Lastly, the nocifensive response in the cold plantar assay did not differ between the hR100E and hWT mice (Fig. 3C), and no difference between male and female mice was observed on the different tests. These results show that the hNGF-R100E mutation does not have an impact on normal physiological responses to mechanical, heat, and cold stimulation.

### 3.3. Impact of hNGF-R100E mutation on pain-related behavior in chemical and inflammation-induced pain models

To evaluate the effect of hNGF-hR100E mutation on pain-like behavior that develops in response to injection of inflammatory agents, we used the formalin and CFA assays. In the formalin assay, the male hNGF-R100E and hNGF-WT mice developed similar degrees of nocifensive behaviors during the first and second phase of the model, both when compared across time points (Fig. S2A, http://links.lww.com/PAIN/C98) and when the area under the curve was calculated (Fig. S2B, C, http://links.lww.com/PAIN/C98), indicating that hNGF-R100E mice maintain intact responsiveness to this type of chemically induced acute nociception.

**Figure 4.**
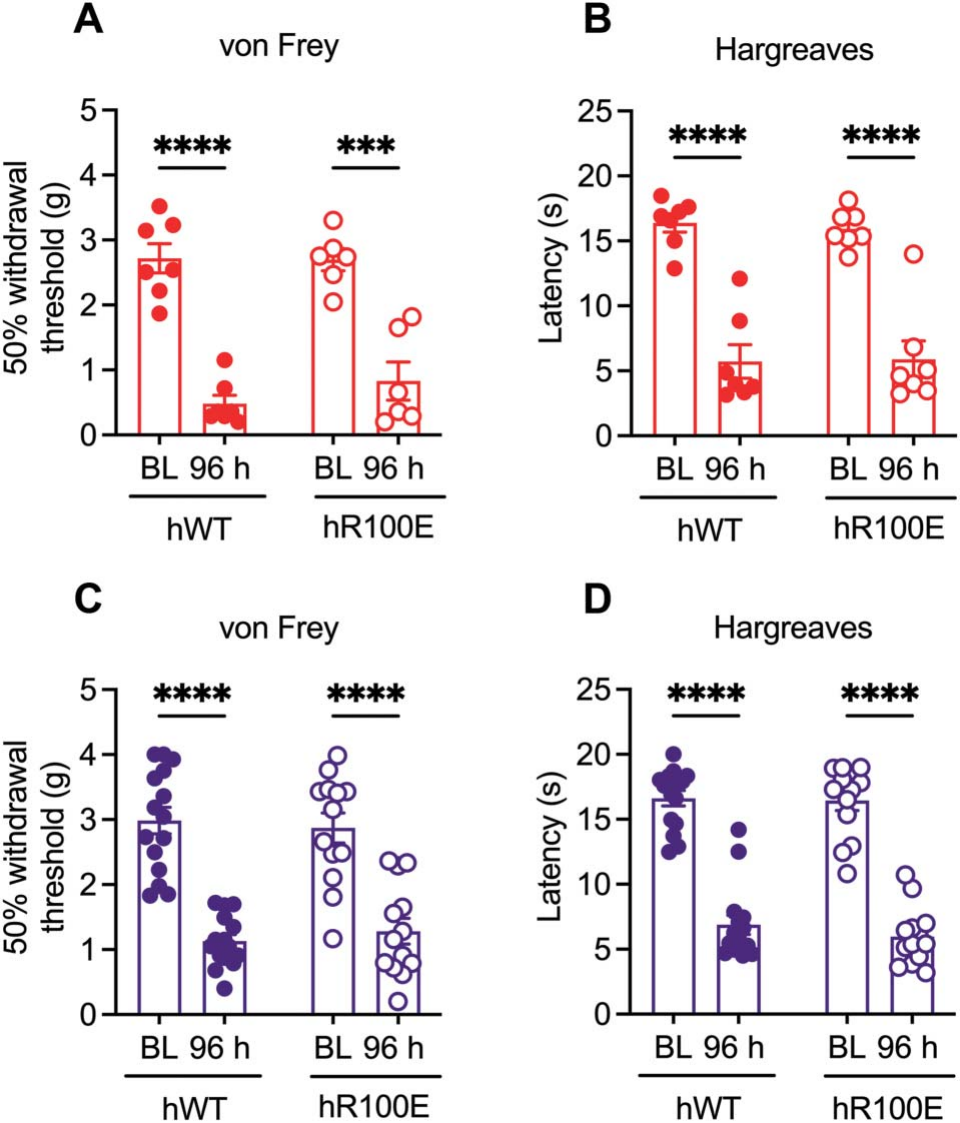
The hNGF-R100E mutation does not prevent mechanical and thermal hypersensitivity after CFA injection into the ankle joint. Four days after the intra-articular CFA injection, female (red) and male (purple) hNGF-WT and hNGF-R100E mice developed the same degree of hypersensitivity to mechanical (A and C) and heat (B and D) stimulus measured using von Frey filaments and the modified Hargreaves box, respectively. Data are presented as mean ± SEM. Each dot represents one mouse. For mechanical thresholds, n = 6 to 7 females and 13 to 15 males/group; heat thresholds, n = 7 females and 7 to 8 males/group. ***P = 0.0001; ****P < 0.0001 by 2-way ANOVA and Bonferroni post-hoc. CFA, complete Freund adjuvant.

To investigate the response to joint inflammation, we administered CFA into the ankle joint of hNGF-R100E and hNGF-WT mice. On day 3 post-CFA injection, we found similar response to the application of acetone in the paw of female hNGF-WT and hNGF-R100E mice (Fig. S2D, http://links.lww.com/PAIN/C98). Four days after the CFA injection, we detected similar mechanical and heat hypersensitivity levels in hNGF-WT and hNGF-R100E female (Fig. 4A, B) and male mice (Fig. 4C, D). A similar degree of spontaneous behaviors was observed between hNGF-WT and hNGF-R100E female mice (Fig. S2E, http://links.lww.com/PAIN/C98) at 24, 72, and 96 hours after the CFA injection. However, hNGF-WT male mice showed a progressive increase in the time of spontaneous guarding, lifting, and licking of the injected leg, which was not observed in the hNGF-R100E mice (Fig. S2F, http://links.lww.com/PAIN/C98). When the magnitude of the spontaneous behaviors was calculated by area under the curve, it was found to be significantly higher in hNGF-WT male mice compared with hNGF-WT females and hNGF-R100E males and females (Fig. S2G, http://links.lww.com/PAIN/C98). These findings suggest that the mechanisms of skin sensitization induced by inflammation in the ankle joint remain functional in hNGF-R100E mice.

### 3.4. Sex-dependent gene expression on the inflamed ankle joints of hNGF-R100E mice

**Figure 5.**
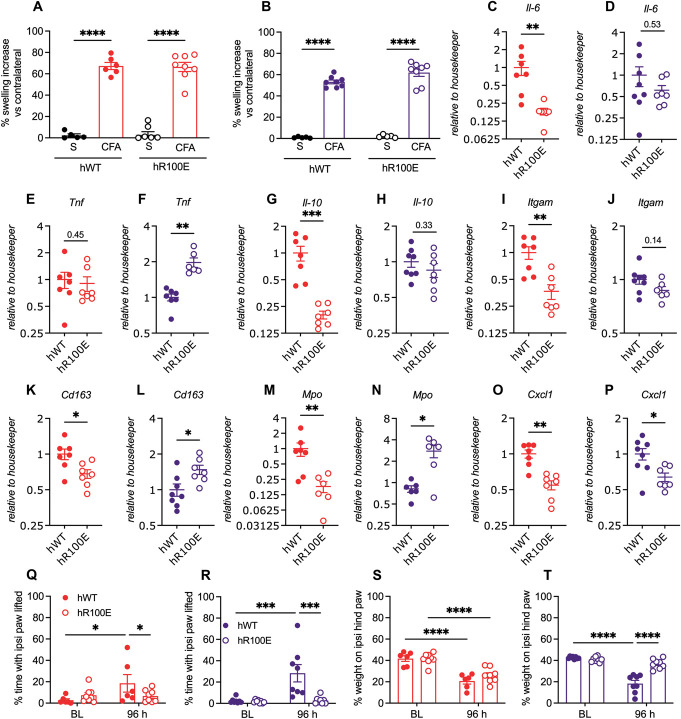
NGF and sex-dependent changes in weight bearing and mRNA expression after the CFA injection into the ankle joint. The injection of CFA into the ankle joint induces a similar degree of inflammation in both hNGF-WT and hNGF-R100E female (A) and male (B) mice. Expression of *Il-6* was significantly lower in female (C) but not in male (D) hNGF-R100E mice compared with hNGF-WT. No changes on *Tnf* mRNA in hNGF-R100E females (E) compared with hNGF-WT female mice. The mRNA expression levels of *Tnf* were significantly increased in hNGF-R100E males (F) compared to their hNGF-WT counterparts. *Il-10* mRNA levels were decreased in female (G), but not altered in male (H) hNGF-R100E mice. Similarly, *Itgam* mRNA levels decreased in female (I) but did not change in male (J) hNGF-R100E mice. *Cd163* mRNA was significantly decreased in female (K) and elevated in male (L) hNGF-R100E mice. In a similar fashion, *Mpo* mRNA was significantly decreased in female (M) and elevated in male (N) hNGF-R100E mice. The mRNA levels of *Cxcl1* were significantly decreased in both female (O) and male (P) hNGF-R100E mice. Weight bearing was assessed before and 96 hours after CFA injection. Both female and male hNGF-WT showed a significant increase in the time of protection of the injected ankle, but female (Q) and male (R) hNGF-R100E mice did not display changes regarding baseline values. A significant decrease in the weight borne by the injected ankle was found in hNGF-WT and hNGF-R100E females (S) and hNGF-WT males but not in hNGF-R100E males (T). Data are presented as mean ± SEM. Each dot represents one mouse, n = 5 to 8 mice/group. *P < 0.05; **P < 0.01; ***P < 0.001; ****P < 0.0001 by unpaired Mann–Whitney, or 2-way ANOVA and Bonferroni post-hoc. CFA, complete Freund adjuvant; NGF, nerve growth factor.

Intra-articular injection of CFA into the ankle joint resulted in a marked swelling of the joint in both hNGF-WT and hNGF-R100E females (Fig. 5A) and male (Fig. 5B) mice compared to the control groups receiving i.a. injection of saline. Intriguing patterns emerged when we examined the differences between hNGF-R100E and hNGF-WT mice mRNA levels on the inflamed joints. In hNGF-R100E female mice, we observed a significant reduction in the mRNA levels of *Il-6* (Fig. 5C), but it was not significant in male mice (Fig. 5D). *Tnf* mRNA levels were unaltered in hNGF-R100E female mice (Fig. 5E) but significantly elevated in male mice (Fig. 5F). In addition, *Il-10* mRNA levels were significantly lower in female (Fig. 5G) and unaltered in male (Fig. 5H) mice.

When comparing macrophage-related factors, we found that *Itgam* mRNA levels were significantly lower in hNGF-R100E females (Fig. 5I), but no changes in males (Fig. 5J) were found. Surprisingly, we found that the expression of *Cd163* in hNGF-R100E females was significantly lower (Fig. 5K) and higher in males (Fig. 5L) compared with their hWT-NGF counterparts. To investigate changes in mRNA levels associated with neutrophils, we assessed *Mpo* levels. Surprisingly, we discovered significantly lower *Mpo* mRNA levels in hNGF-R100E females compared to their hNGF-WT counterparts (Fig. 5M). Conversely, *Mpo* mRNA levels were significantly elevated in hNGF-R100E males compared to hNGF-WT males (Fig. 5N). Lastly, we observed a significant decrease in *Cxcl1* mRNA levels for both female (Fig. 5O) and male (Fig. 5P) hNGF-R100E mice compared to their respective hNGF-WT counterparts.

### 3.5. Weight-bearing tests revealed signs of insensitivity to joint pain in hNGF-R100E mice

Insensitivity to deep joint pain leading to, eg, pain-free fractures has been reported in patients carrying the NGF-R100W mutation.^36^ Therefore, we sought to investigate whether the hNGF-R100E mutation had an impact on joint-related pain in the mice. We determined changes in weight bearing and gait parameters as an outcome measures for pain-related behaviors dependent on joint loading, as a contrast to mechanical stimulation of the skin with von Frey filaments in female and male mice subjected to injection of CFA into the ankle or knee joint.

Complete Freund adjuvant led to a significant increase in the time spent guarding the inflamed paw (Fig. 5Q, R) and reduced weight bearing on the ipsilateral leg (Fig. 5S, T) in hNGF-WT female and male mice. The inflammation caused by CFA did not result in an increase in the duration of guarding the inflamed paw in female (Fig. 5Q) and male (Fig. 5R) hNGF-R100E mice. Furthermore, the reduction in weight bearing on the ipsilateral paw was completely abolished in male hNGF-R100E (Fig. 5S) mice but not in female hNGF-R100E mice (Fig. 5T).

**Figure 6.**
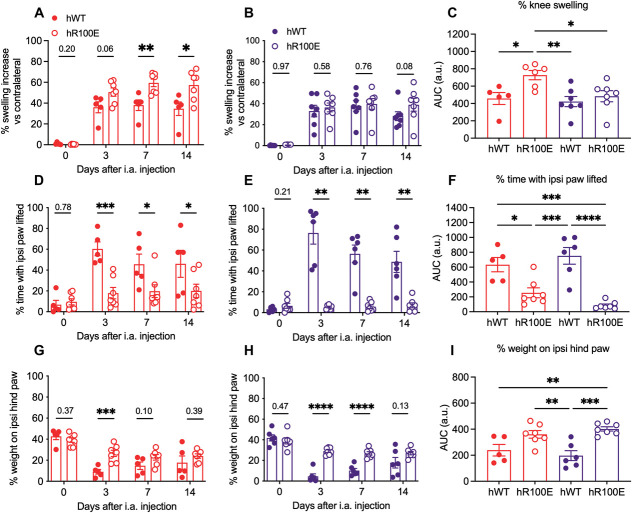
hNGF-R100E mutation protects against DWB impairment but not swelling induced by CFA injection into the knee joint. CFA injection increases knee swelling in both female (A) and male (B) hNGF-R100E and hNGF-WT mice. However, the magnitude was significantly higher in female hNGF-R100E mice compared to hNGF-WT female and male mice (C). The protection of the inflamed leg was significantly lower in female (D) and male (E) hNGF-R100E mice compared with hNGF-WT mice across experimental time points or when the area under the curve was calculated (F). Both female (G) and male (H) hNGF-R100E mice had significantly less weight-bearing impairment than hWT mice, and the magnitude of this change was significantly different (I). Data are presented as mean ± SEM. Each dot represents one mouse, n = 5 to 7 mice/group. *P < 0.05; **P < 0.01; ***P < 0.001; ****P < 0.0001 by one-way ANOVA and Tukey post-hoc, or 2-way ANOVA and Bonferroni post-hoc. CFA, complete Freund adjuvant; DWB, dynamic weight bearing; NGF, nerve growth factor.

The injection of CFA into the knee joint resulted in an increase in knee swelling compared with the noninjected contralateral knee joint (Fig. 6A–C). Surprisingly, this increase was higher in hNGF-R100E female mice at 7 and 14 days after the i.a. injection (Fig. 6A) compared to hNGF-WT female and male mice (Fig. 6C). We did not find a difference in the increase of swelling between hNGF-R100E and hNGF-WT male mice (Fig. 6B) at any tested experimental time point. The time spent guarding the inflamed leg was significantly lower in both female (Fig. 6D) and male (Fig. 6E) hNGF-R100E compared with the hNGF-WT mice. We found no differences between female and hNGF-R100E male mice on the guarding time (Fig. 6F).

Weight-bearing analysis showed that the weight endured on the ipsilateral leg was significantly lower at day 3 post-CFA in hNGF-WT female mice compared with the hNGF-R100E female mice (Fig. 6G) and significantly lower at day 3 and 7 in male mice (Fig. 6H) when compared to hNGF-WT and hNGF-R100E mice. There were no differences between hNGF-R100E female and male mice weight bearing (Fig. 6I). Overall, these results suggest that hNGF-R100E male mice are insensitive to deep joint pain and could continue to use the leg normally despite the presence of joint inflammation, while the mutation did not equally protect female hNGF-R100E mice.

### 3.6. Gait is not affected by inflammation in the ankle or knee joint of hNGF-R100E mice

**Figure 7.**
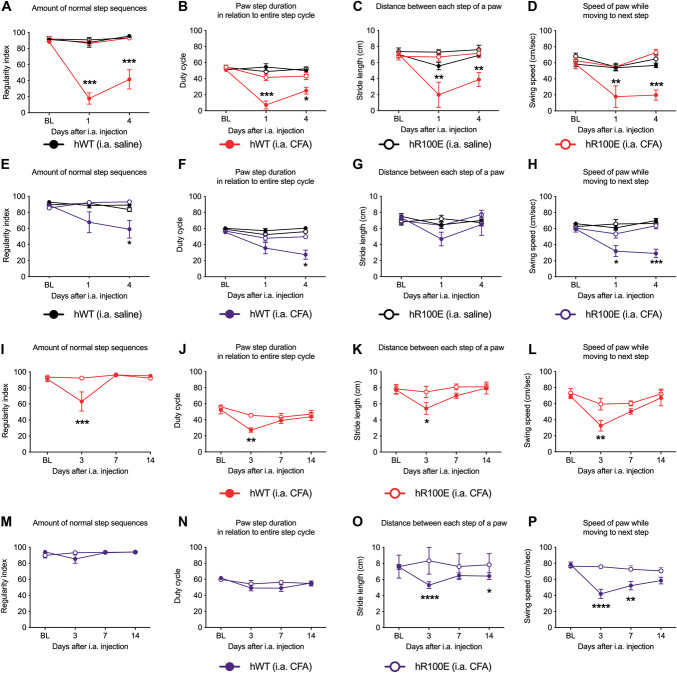
hR100E mice do not show changes in gait after the CFA injection into the ankle or knee joint. When injected into the ankle joint, CFA significantly decreases the regularity index, duty cycle, stride length, and swing speed in female (A–D) hNGF-WT mice at days 1 and 4. Male hNGF-WT mice only showed a significant decrease in the regularity index (E) and duty cycle (F) at day 4, with no changes in the stride length (G), and decreased swing speed (H) at days 1 and 4. hNGF-R100E mice did not develop CFA-induced impairments in gait. Saline-injected female and male hNGF-WT and hNGF-R100E mice were also included as a control, and no signs of gait impairment were detected. Similarly, hNGF-R100E mice did not show changes in gait after CFA injection into in the knee joint. hNGF-WT females showed a significant decrease in the regularity index (I), duty cycle (J), stride length (K), and swing speed (L). No changes were detected in the regularity index (M) or the duty cycle (N) in hNGF-WT male mice, but significantly decreased stride length (O) and swing speed (P) were found. Data are presented as mean ± SEM, n = 5 to 7 mice/group. *P < 0.05; **P < 0.01; ***P < 0.001; ****P < 0.0001 by 2-way ANOVA and Bonferroni post-hoc. CFA, complete Freund adjuvant; NGF, nerve growth factor.

We next assessed different gait parameters in CFA-injected hNGF-WT and hNGF-R100E mice using the CatWalk system to evaluate if the hNGF-R100E mutation protects mice from deep joint nociceptive stimulation. On days 1 and 4 after the CFA injection into the ankle joint, hNGF-WT female mice showed impaired gait, represented by a decrease in the regularity index (Fig. 7A), duty cycle (Fig. 7B), stride length (Fig. 7C), and swing speed (Fig. 7D). On hNGF-WT males, the regularity index (Fig. 7E) and duty cycle (Fig. 7F) were significantly decreased at day 4 after the CFA injection; the stride length was not altered (Fig. 7G), and the swing speed was decreased at both time points (Fig. 7H). In contrast, the female and male hNGF-R100E mice were protected against the CFA-induced changes in the regularity index (Fig. 7A, E), duty cycle (Fig. 7B, F), stride length (Fig. 7C, G), and swing speed (Fig. 7D, H). Control mice injected with saline into the ankle joint did not show changes in any of the analyzed gait parameters in either sex or genotype (Fig. 7A–H).

Injection of CFA into the knee joint to male and female hNGF-WT and hNGF-R100E mice yielded similar, but somewhat milder, changes in the 4 gait parameters compared to what was recorded after ankle joint injection of CFA. The hNGF-WT, but not hNGF-R100E female mice, injected with CFA into the knee joint displayed a significant decrease in the 4 analyzed parameters on day 3 (Fig. 7I–L). The hNGF-WT, but not hNGF-R100E male mice, showed no changes in the regularity index (Fig. 7M) or duty cycle (Fig. 7N) but a significant decrease on day 3 and 14 on the stride length (Fig. 7O) and the swing speed on days 3 and 7 (Fig. 7P).

Taken together, these data indicate that hNGF-R100E mice are deficient in the sensitization mechanism of the inflamed joint. As a result of this, both female and male mice carrying the hNGF-R100E mutation did not adjust their gait, even in the presence of inflammation in the ankle or knee joint, suggesting altered nociceptive processing compared with the hNGF-WT mice.

### 3.7. The hNGF-R100E mutation does not reduce complete Freund adjuvant–induced nerve growth in the synovium

**Figure 8.**
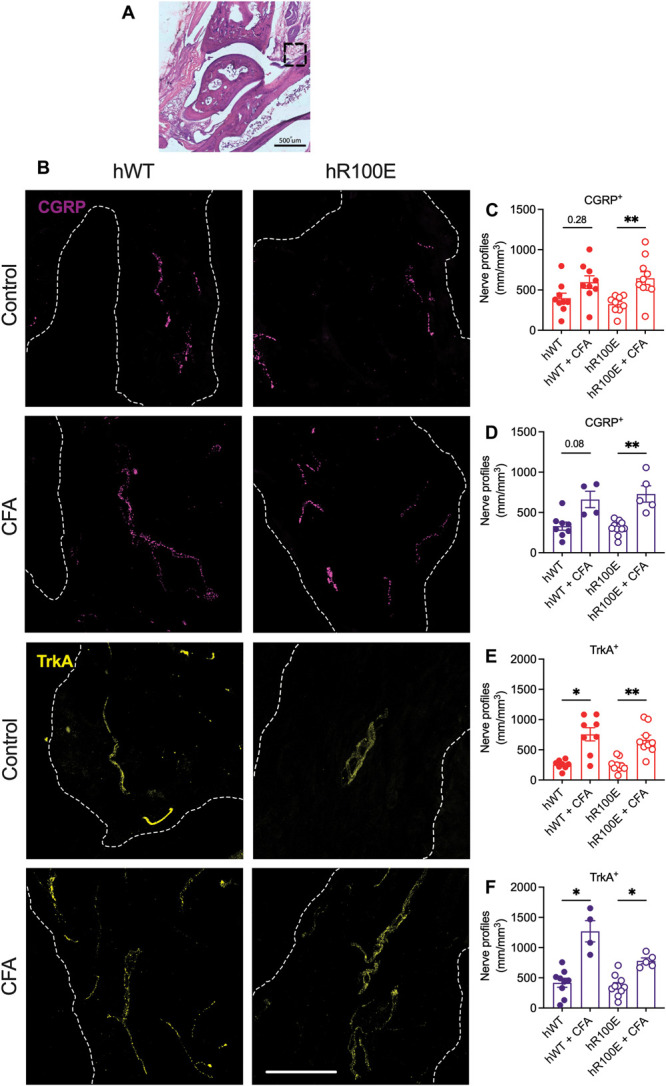
The hR100E mutation does not have an impact on the CFA-induced nerve sprouting. Representative images showing the synovial innervation (A) of CGRP- and TrkA-positive nerve profiles in hWT and hR100E female mice without inflammation and after the CFA injection (B). After the CFA injection into the ankle joint, no significant changes were found in the density of CGRP nerve profiles in hWT female or male mice (C and D); however, they were significantly increased in hR100E female and male mice. On the other hand, the TrkA nerve profiles were increased in both hWT and hR100E female (E) and male (F) mice. Data are presented as mean ± SEM. Each dot represents one mouse, n = 4 to 11 mice/group. The white segmented line delimits the synovium. Scale bar = 50 μm. *P < 0.05, **P < 0.01 by unpaired *t* test. CFA, complete Freund adjuvant; CGRP, calcitonin gene-related peptide.

To explore if the hNGF-R100E mutation impacts the innervation of inflamed joints, we quantified the density of CGRP and TrkA nerve profiles in the talus-tibia synovium (Fig. 8A, B). Our results only showed a tendency to increase in the density of CGRP-positive nerve profiles in hNGF-WT female and male mice while the CGRP nerve profile density was significantly elevated in both hNGF-R100E female and male mice (Fig. 8C, D). TrkA-positive nerve profile density was significantly increased after the CFA injection in hNGF-WT and hNGF-R100E female and male mice (Fig. 8E, F).

These data indicate that under inflammatory conditions, nerve sprouting is not altered by the hNGF-R100E mutation, and that in the case of the hNGF-R100E female and male mice, the insensitivity to joint stimulation cannot be explained by decreased nerve sprouting.

### 3.8. Female hNGF-R100E mice are protected from complete Freund adjuvant–induced bone erosion

**Figure 9.**
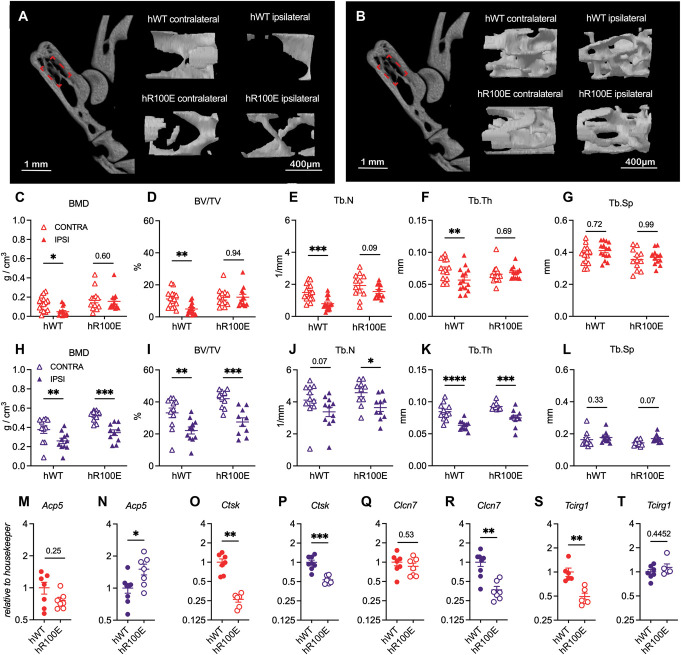
hR100E female mice are protected from CFA-induced bone loss. The calcaneus from hNGF-WT and hNGF-R100E female (A) and male (B) mice were analyzed by microCT. CFA induces a significant decrease in BMD (C), BV/TV (D), Tb.N (E), and Tb.Th (F) but no changes in Tb.Sp (G) in hNGF-WT mice. The BMD (H), BV/TV (I), and Tb.Th (K) were decreased in hNGF-WT male mice, but no change in Tb.N (J) or Tb.Sp (L) were found. The bone parameters of hNGF-R100E female mice remained unaltered after the CFA injection, but hNGF-R100E male mice developed similar bone changes to hNGF-WT mice (H–L). In the joint of hNGF-R100E, the mRNA levels of *Acp5* were not altered in females (M) but were significantly elevated in males (N); *Ctsk* levels were decreased in both females (O) and males (P); *Clncn7* was unaltered in females (Q) but lower in males (R); *Tcirg1* levels were significantly lower on females (S) but unaltered on males (T). Filled triangles represent the ipsilateral side, and empty triangles the contralateral side (C–L). Data are presented as mean ± SEM. Each dot represents one mouse, n = 10 to 15 mice/group. *P < 0.05; **P < 0.01; ***P < 0.001; ****P < 0.0001 by unpaired *t* test or 2-way ANOVA. BMD, bone mineral density; BV/TV, trabecular bone volume to total volume fraction; Tb.N, trabecular number; Tb.Sp, trabecular separation; Tb.Th, trabecular thickness; μCT, microcomputed tomography. CFA, complete Freund adjuvant; NGF, nerve growth factor.

Finally, to explore if the hNGF-R100E mutation influences CFA-induced bone erosion, ankle (Fig. 9A, B) and knee joints were analyzed by microCT. The results showed that 14 days after the CFA injection into the ankle joint, hNGF-WT female mice had a significant decrease in BMD (Fig. 9C), BV/TV ratio (Fig. 9D), Tb.N (Fig. 9E), and Tb.Th (Fig. 9F) in the calcaneus, but not changes in the Tb.Sp (Fig. 9G). Similar changes were found in hNGF-WT male mice (Fig. 9H–L). Notably, hNGF-R100E female mice did not show changes in any analyzed parameter after the CFA injection (Fig. 9C–G). Analysis of calcaneus from hNGF-R100E male mice showed that the BMD (Fig. 9H), BV/TV ratio (Fig. 9I), Tb.N (Fig. 9J), and Tb.Th (Fig. 9K) were significantly decreased while no change in Tb.Sp (Fig. 9L) was identified after CFA injection compared with hNGF-WT mice. In addition, when we analyzed mRNA levels in the ankle joint, we found no changes in *Acp5* mRNA levels in hR100E female mice (Fig. 9M) and increased levels in male mice (Fig. 9N). We found significantly lower levels of *Ctsk* in both female (Fig. 9O) and male (Fig. 9P) hNGF-R100E mice. We also investigated whether the mRNA levels of factors related to osteoclast chloride channels (*Clcn7*) and osteoclast vacuolar proton pump (*Tcirg1*) were altered by the R100E mutation under inflammatory conditions. Our results showed that *Clcn7* were not different in hNGF-R100E females (Fig. 9Q); however, they were significantly lower in hNGF-R100E males (Fig. 9R). Furthermore, *Tcirg1* levels were significantly lower in hNGF-R100E females compared with hNGF-WT females (Fig. 9S). On hNGF-R100E males, no change in *Tcirg1* was found (Fig. 9T) when compared with hNGF-WT male mice.

Similarly, when other bones in the ankle joint were analyzed, we found that female and male hNGF-WT mice developed bone loss in the talus after the CFA injection (Fig. S3, http://links.lww.com/PAIN/C98), and only female hNGF-R100E mice were protected against the CFA-induced bone loss (Fig. S3A-E, http://links.lww.com/PAIN/C98), while male hNGF-R100E mice were not protected and developed similar bone loss compared with the male hNGF-WT mice (Fig. S3F-J, http://links.lww.com/PAIN/C98). Interestingly, when the distal tibia was analyzed, no signs of bone loss were found on either female or male hNGF WT or hNGF-R100E mice (Fig. S4A-L, http://links.lww.com/PAIN/C98).

When the CFA was injected into the knee joint, we found no signs of bone erosion neither in the distal femur (Fig. S5A-P, http://links.lww.com/PAIN/C98) nor in the proximal tibia (Fig. S6A-P, http://links.lww.com/PAIN/C98) of female or male hNGF-WT or hNGF-R100E mice.

These results indicate that while the hNGF-R100E mutation prevents CFA-induced bone loss in female mice, it does not have a similar effect in male mice. In addition, the CFA-induced bone loss affected small bones, such as the calcaneus and talus, but not large bones, such as the distal femur or tibia. This might be coupled with the more prominent change in weight bearing and gait when CFA was injected into the ankle joint.

## 4. Discussion

Researchers investigating congenital insensitivity to pain disorders have made a significant breakthrough by identifying a single-point mutation in the NGFβ gene. This discovery has led to the development of mutated painless NGF for potential therapeutic applications in various disease areas.^11,12^ Using a knock-in mouse model carrying the sequence encoding human NGF-R100E, we examined its impact on pain perception and bone microstructure. Our findings reveal that the mutation does not affect the development of the sensory nervous system or skin sensitivity to mechanical, heat, or cold stimuli in naïve animals and after intra-articular injection of CFA. However, it offers protection against deep joint loading–dependent pain-like behaviors in female and male mice with CFA-induced arthritis. Interestingly, only female mice with the mutation were protected against bone erosion caused by joint inflammation.

Nerve growth factor mutation may cause insensitivity to joint pain by several distinct mechanisms. Nerve biopsies from patients with Norrbottnian HSAN V revealed a marked reduction in the number of unmyelinated C- and lightly myelinated Aδ fibers, suggesting the involvement of developmental abnormalities in the nociceptive nervous system.^37^ Similarly, heterozygous NGF^R100W/m^ mice showed an age-dependent reduction in skin innervation.^52,53,58^ On the contrary, our immunohistochemical data from hNGF-R100E homozygous mice demonstrated that the development of the nociceptive nervous system was comparable to hNGF-WT mice in the L5 DRG, sciatic nerve synovium and glabrous skin. Supporting our finding, human fetal DRG neurons exposed to hNGF-R100E displayed neurite outgrowth.^17^

Previous research indicated that the hNGF-R100W, but not the hNGFR100E, mutation affects NGF processing and release, with notably low production yields in vitro.^31^ This discrepancy suggests that the impact of the mutation on the processing and secretion of the hNGF-R100E protein is less pronounced, leading to sufficient availability of NGF for survival and differentiation of nociceptive neurons. Therefore, it is likely that the lack of CFA-induced changes in joint loading–dependent behavior in the hNGF-R100E mice is mainly attributed to impairment in NGF-mediated intracellular signaling rather than a reduced NGF secretion or impaired innervation.

Development of pain-like behaviors and nerve sprouting of CGRP-expressing nerve fibers have been previously reported in mice after injection of CFA into the knee joint.^22^ However, we only found a tendency to increased density of CGRP-positive nerve profiles detected in hNGF-WT female and male mice post-CFA. This discrepancy may be explained by the differences in location (ankle vs knee) and duration (our study was shorter). Interestingly, in hNGF-R100E female and male mice, the number of CGRP-positive nerve profiles was significantly increased after CFA injection. This finding demonstrates that the R100E mutation does not limit the sensory nervous system capacity to react to trophic factors released in the ankle joint. Sprouting of CGRP-positive nerve fibers has been associated with more severe pain-like behaviors.^22,27^ We did not find such a relationship, as the R100E female and male mice showed reduced changes in joint loading–dependent parameters, despite a statistically significant increase of CGRP-positive nerve profiles in the synovium.

Recent work has demonstrated that the TrkA antagonist AR786 reduces pain-like behaviors in rodent models of inflammatory arthritis^5^ and osteoarthritis.^41^ The density of TrkA-positive nerve profiles was increased in both female and male mice of both genotypes in response to joint inflammation in our study, yet the R100E mice displayed reduced alterations in joint loading–dependent behaviors. Thus, the observed insensitivity to deep tissue stimulation in the hNGF-R100E mutated mice can also not be rooted in reduced sprouting of TrkA-positive fibers.

Injection of CFA induces swelling, immune cell recruitment, and nerve sprouting. The levels of mature NGF increase at the site of inflammation both after injection of CFA and carrageenan.^2,33^ It has been shown that different human and murine cell types can produce and release NGF, including epithelial cells, fibroblast-like synoviocytes, chondrocytes, and macrophages in the joints,^7,44,51^ contributing to the development and maintenance of inflammation. Our data showed that *Tnf*, *Il6*, and *Il10* mRNA was differentially regulated in the inflamed joints of mutated R100E mice compared with the hNGF-WT mice, and this was sex-dependent. In addition, we observed a higher magnitude of CFA-induced swelling in the knee joint of females compared to male hNGF-R100E mice. Interestingly, previous reports showed that the neutralization of NGF in a model of experimental autoimmune encephalomyelitis increases tissue inflammation in female rats.^35^ In addition, treatment of female rabbits subjected to surgically induced osteoarthritis with an anti-NGF antibody resulted in an increased diameter of the affected joints, while pain-like behaviors decreased.^34^ This suggests that, at least in female mice and rabbits, the role of NGF in pain-like sensitization and inflammation may go in opposing directions. However, the exact mechanisms through which mutated NGF or NGF-neutralization promotes inflammation in females is unclear and warrants further investigation.

As expected, CFA-induced joint inflammation leads to bone loss, which has also been reported in animal models of joint inflammation.^3^ This is also common in human conditions with chronic joint inflammation, such as rheumatoid arthritis.^43,46^ In pathological conditions, overexpression of certain molecules, such as IL-6, TNF, histamine, and NGF, in the joints can promote osteoclast overactivity and diminished osteoblast activity. For instance, it has been shown that IL-6 can increase the expression of RANKL, which stimulates maturation and increases the activity of osteoclasts.^55^ Our study found that the hNGF-R100E mutation prevented bone loss induced by joint inflammation in female mice, while swelling was not downregulated. In contrast, hNGF-R100E male and hNGF-WT female and male mice developed signs of bone loss induced by the CFA injection. Sex dimorphism in bone metabolism is well documented; for instance, the enhanced response to RANKL by osteoclasts from female mice and its association with an increased cell number has been reported.^38^ Interestingly, we found that some osteoclast genes are differentially regulated in the hNGF-R100E mice, and this was sex-dependent. Thus, an altered osteoclast activity might explain the protection against bone loss in the hNGF-R100E female mice. This discovery suggests that altering the interaction between NGF and its receptors, as seen in female mice with hR100E-NGF, could potentially provide therapeutic benefits for managing chronic pain and preventing osteoporosis. This is especially relevant in cases such as osteoarthritis and rheumatoid arthritis, where female patients are more susceptible to such conditions.^13,45,54^

Our results showed that the R100E mutation does not modify the response to mechanical and thermal stimulation after CFA injection in female or male mice. Importantly, the hNGF-R100E male mice did not show signs of joint-related pain-like behaviors, and the magnitude in female mice was lower than in their hWT counterpart. This discrepancy between superficial and deep sensitivity, ie, skin vs joints, after development of joint inflammation in hR100E mice is noteworthy. One possible explanation for this disparity is the difference in the innervation pattern between the skin and bone/joint. Approximately 30% of the sensory nerve fibers innervating skin are TrkA immune-positive. In comparison, more than 80% of the sensory nerve fibers innervating bone/joint are TrkA positive,^14^ suggesting the predominant role of NGF-dependent mechanisms in the sensitization process in the bone/joint.

Nerve growth factor exerts its actions via the high-affinity TrkA and the low-affinity p75NTR receptors. Nerve growth factor-TrkA signaling is associated with neuronal survival, differentiation, and synaptic plasticity while p75NTR activation can enhance neuronal survival by promoting TrkA signaling or induce cell apoptosis. The NGF-R100E mutation drastically reduce the affinity to p75-NTR, while the binding to TrkA remains preserved,^11^ suggesting that the mutation on R100 selectively impacts the interaction between NGF and p75-NTR. Because activation of the p75-NTR signaling pathway can contribute to the nociceptive sensitization,^42,57^ the reduced p75-NTR signaling upon inflammation-induced NGF-R100E release, compared to NGF release in WT mice, may explain the insensitivity to stimulation of deep tissues and the consequent normal use of the inflamed joint. While the role of the NGF-TrkA interaction in pain is well established, fewer attempts have been made to elucidate the involvement of the NGF-p75-NTR interaction in the pathogenesis of inflammatory pain. Given that the binding to p75-NTR is selectively altered in hR100E NGF,^11^ these findings indicate that hNGF-R100E mice may serve as a model that can delineate the specific role of the NGF-p75-NTR interaction in pathological pain processes.

In conclusion, our data showed that hNGF-R100E does not impact the development of the sensory nervous system or alter the development of punctate mechanical and thermal sensitivity on the skin under naïve or inflammatory conditions. However, the hNGF-R100E mutation provides protection against deep joint pain in both male and female mice subjected to unilateral arthritis. Besides this, the hNGF-R100E mutation has a protective effect against bone loss induced by inflammation in female mice. Thus, it suggests that the NGF-R100E mutation alters the development of joint pain specifically without compromising other sensory functions and, in females, without an impact on bone microarchitecture. This finding emphasizes the importance of in-depth elucidation, and the modulation of the NGF signaling, rather than NGF-blockade, could offer a potential avenue for pain relief and attenuation of neurodegenerative diseases without the bone-related side effects observed in anti-NGF trials.

## Conflict of interest statement

The authors have no conflicts of interest to declare.

## Appendix A. Supplemental digital content

Supplemental digital content associated with this article can be found online at http://links.lww.com/PAIN/C98.

## Supplementary Material
