# Supplementary material for: Sex-dependent effects of the targeted nerve growth factor mutation (R100E) on pain behavior, joint inflammation, and bone erosion in mice
Source: Pain. 2024 Sep 25;165(12):2814–28. doi: 10.1097/j.pain.0000000000003343 (PMC11562760; doi:10.1097/j.pain.0000000000003343)
Supplement: Supplementary file 1 [file jop-165-2814-s001.pdf]

Supplementary methods

Formalin test

Under isoflurane anesthesia, 10 µl of 5% formalin in saline was injected subcutaneously into the dorsal part of one hind paw [1; 2]. The animals were then placed into acrylic chambers (13 x 13 cm) resting on a pane of polycarbonate floor. The animals were filmed from the front with a tilted mirror placed below the chambers to allow recording both from the side and underneath the mice. The time spent licking, biting, and shaking the injected paw was measured in 12 blocks of 5 minutes. The group difference was analyzed by comparing the area under the time-response curve (AUC) approximated by the trapezoid rule.

Acetone test

For the acetone drop method, after the habituation in acrylic chambers (13 x 13 x 13 cm) placed on a wire mesh grid floor, an acetone droplet of approximately 10 µl was applied to the plantar surface of one hind paw. The time spent reacting to the acetone (e.g., licking, biting, or shaking the paw) was measured over 60 seconds. The test was repeated three times for each paw, and the mean value was calculated. The baseline sensitivity pre-CFA was assessed on three separate occasions with 1-2 days apart. The day 3 post-CFA sensitivity assessment was performed similarly.

Spontaneous pain-like behaviors

To evaluate spontaneous pain-like behaviors, the mice were acclimatized to the acrylic enclosure (13 x 13 x 13 cm) placed on a glass surface for 60 minutes. Subsequently, the frequency of guarding, lifting, and licking of the affected joint was

quantified using a digital chronometer over a 2-minute observation period. One baseline was assessed 1-2 days before the CFA injection. The spontaneous pain-like behaviors were assessed 24, 72, and 96 hours after the CFA injection.

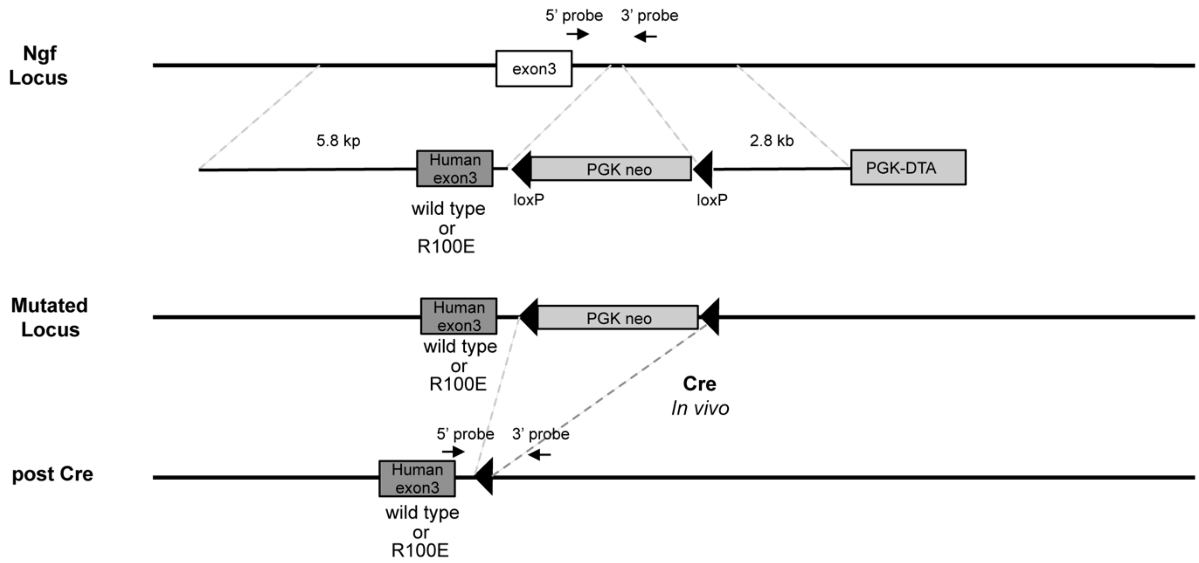

Fig S1: Generation of knock-in mice expressing humanized NGF or human R100E NGF mutation.

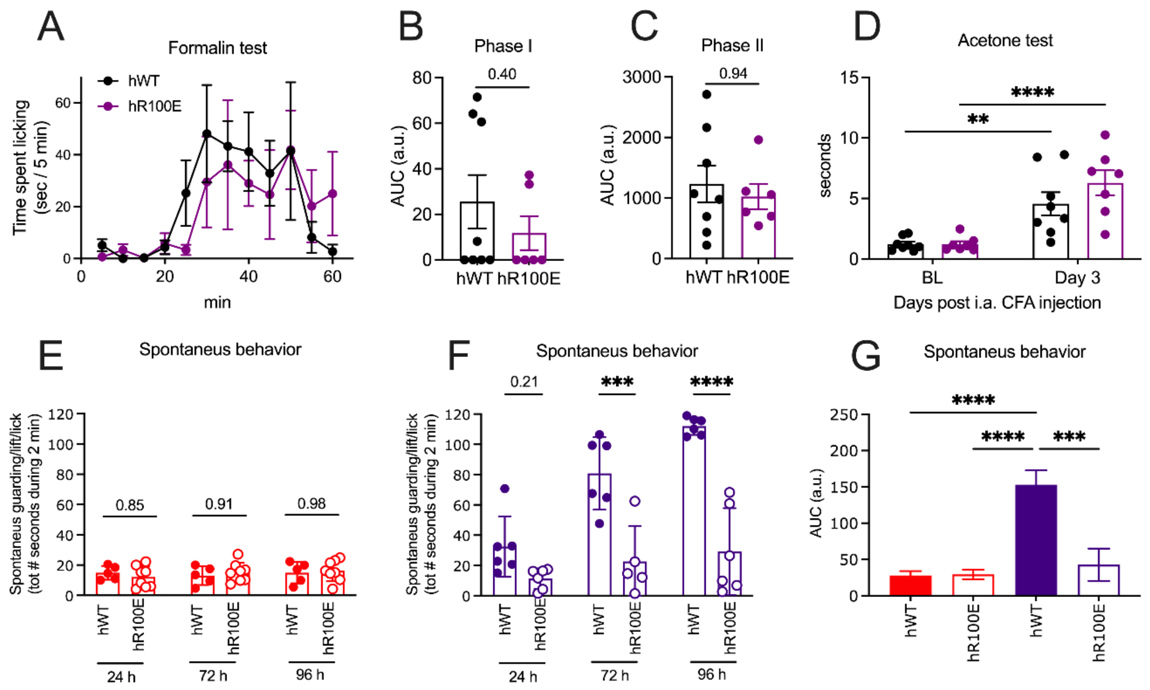

Fig S2. The hNGF-R100E mutation does not alter the responsiveness to chemical or CFA-induced nociception but decreases spontaneous behaviors in male mice. Time spent licking following intradermal injection of formalin into the dorsal part of the hind paw in hNGF-WT and hNGF-R100E male mice is shown in (A). The area under the curve (AUC) for the cumulative nocifensive responses during Phase I (0-10 min) (B) and Phase II (10-60 min) (C) following formalin did not differ between hNGF-WT and hNGF-R100E mice. Similarly, after CFA injection, both hNGF-WT and hNGF-R100E female mice developed the same level of response to the application of acetone drops onto the skin (D). Finally, the time of spontaneous behaviors after CFA recorded in a 2-min period was similar in female mice from both genotypes (E) but significantly lower in hNGF-R100E compared with hNGF-WT male mice (F). AUC analysis showed that spontaneous behavior magnitude was significantly higher in hNGF-WT male mice than in hNGF-WT females and hNGFR-100E (G). Data are presented as mean ± SEM, and 6-8 mice were included in each group. ** $P < 0.01$; *** $P < 0.001$; **** $P < 0.0001$ by one-way ANOVA and Tukey´s post-hoc, or 2-way ANOVA and Bonferroni´s post-hoc.

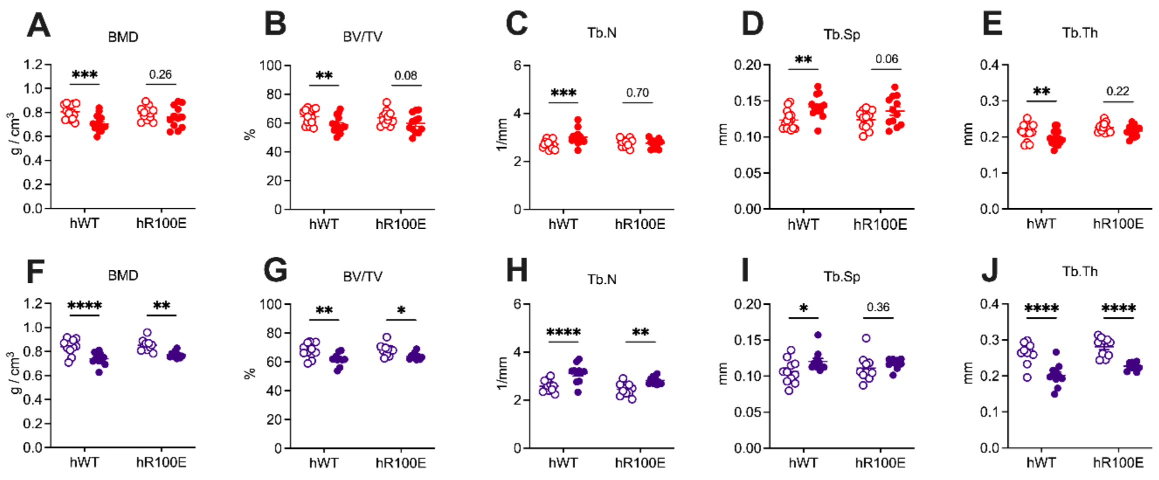

Fig S3. Talus of hR100E female mice is protected from CFA-induced bone loss. CFA induces a significant decrease in BMD (A, F), BV/TV (B, G), Tb.N (C, H), Tb.Sp (D, I) and Tb.Th (E, J) in both female and male hNGF-WT mice. The bone parameters of hNGF-R100E female mice remained unaltered after the CFA injection, but hNGF-R100E male mice developed similar bone changes to hNGF-WT mice. No changes in Tb.Sp was found on any hNGF-R100E male mice (I). Data is presented as mean ± SEM. Each dot represents one mouse, and 10-15 mice were included in each group. * P < 0.05; ** P < 0.01; *** P < 0.001; **** P <0.0001 by 2-way ANOVA. BMD: bone mineral density; BV/TV: trabecular bone volume to total volume fraction; Tb.N: trabecular number; Tb.sp: trabecular separation; Tb.Th: trabecular thickness.

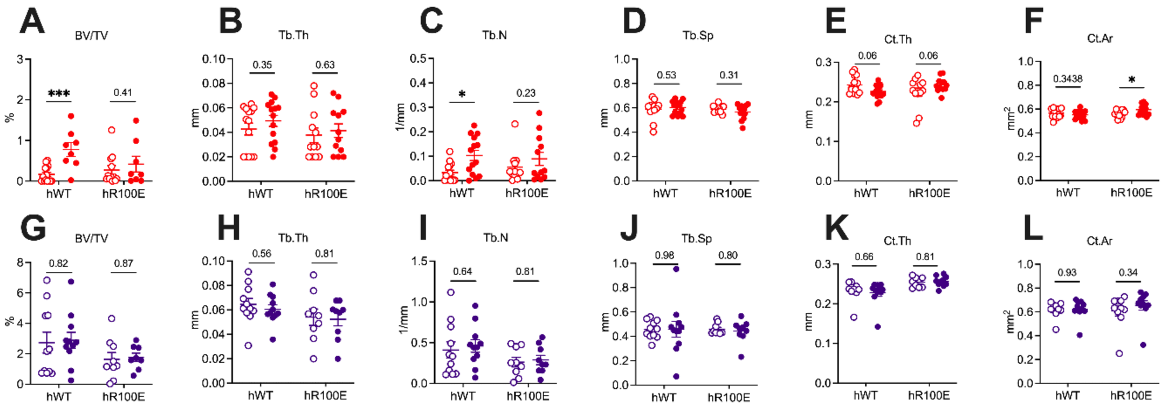

Fig S4. The distal tibia is not affected by CFA. Fourteen days after the CFA injection a significant increase in the BV/TV (A) and Tb.N (C) in hNGF-WT, and an increase or Ct.Ar (F) in hNGF-R100E female mice were found. No changes in Tb.Th (B), Tb.Sp (D), Ct.Th (E) in female mice. In males, the BV/TV (G), Tb.Th (H), Tb.N (I), Tb.sp (J), Ct.Th (K) and Ct.Ar (L) were similar between HNGF-WT and hNGF-R100E mice. Data is presented as mean ± SEM. Each dot represents one mouse, and 8-14 mice were included in each group. * $P < 0.05$; *** $P < 0.001$ by 2-way ANOVA. BV/TV: trabecular bone volume to total volume fraction; Tb.Th: trabecular thickness; Tb.N: trabecular number; Tb.Sp: trabecular separation; Ct.Th: cortical thickness; Ct.Ar: cortical area.

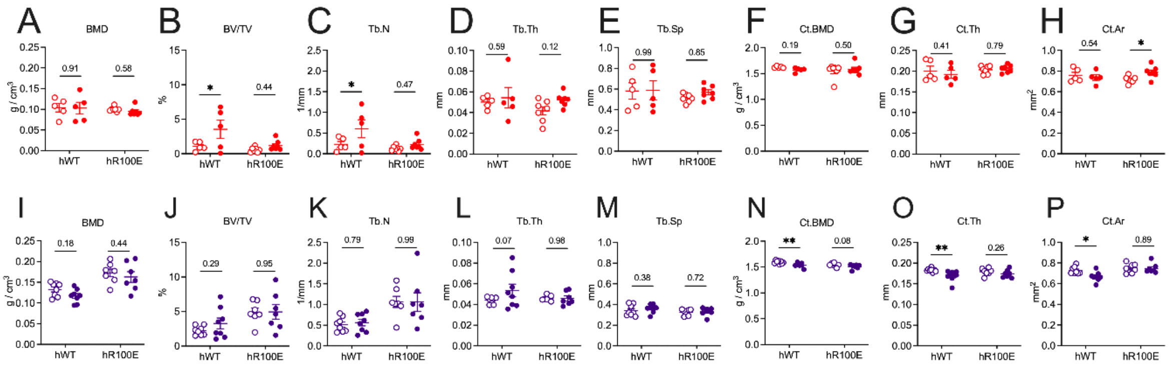

Fig S5. The CFA injection in the knee induces mild changes in the distal femur microarchitecture in hNGF-WT or hNGF-R100E mice. Fourteen days after the CFA injection, we found no changes in BMD (A, I), BV/TV (B, J), Tb.N (C, K), Tb.Th (D, L), Tb.sp (E, M), Ct.BMD (F, N), CT.Th (G, O) on female or male hNGF-R100E mice. Additionally, the Ct.Ar on hNGF-R100E female mice (H) was significantly higher, but not in hNGF-R100E males (P). A higher BV/TV, Tb.N was found in hNGF-WT female mice. The three cortical parameters, Ct.BMD, Ct.Th, and Ct.Ar were decreased in hNGF-WT male mice. Data is presented as mean ± SEM. Each dot represents one mouse, and 5-8 mice were included in each group. * $P < 0.05$; ** $P < 0.01$ by 2-way ANOVA. BV/TV: trabecular bone volume to total volume fraction; Tb.Th: trabecular thickness; Tb.N: Trabecular number; Tb.sp: trabecular separation; Ct.BMD: cortical bone mineral density Ct.Th: cortical thickness; Ct.Ar: cortical area.

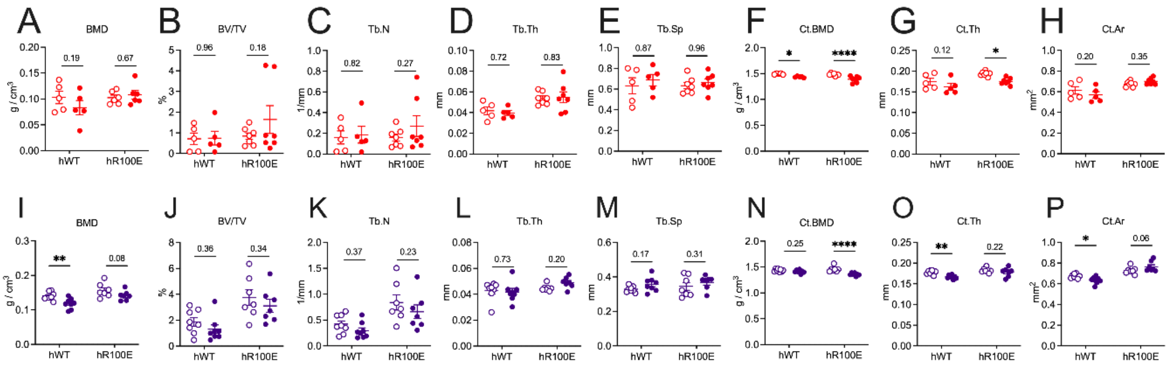

Fig S6. The CFA injection in the knee induces mild changes in the proximal tibia microarchitecture in hNGF-WT or hNGF-R100E mice. Fourteen days after the CFA injection, we found no changes in BMD (A, I), BV/TV (B, J), Tb.N (C, K), Tb.Th (D, L), Tb.Sp (E, M), or Ct.Ar (H, P) on female or male hNGF-R100E mice. A significant decrease in CT.BMD was found in both female (F) and male (N) hNGF-R100E mice. The Ct.Th was decreased on hNGF-R100E but not on hNGF-WT female mice (G); on the contrary, only hNGF-WT males showed lower Ct.Th (O). Data is presented as mean ± SEM. Each dot represents one mouse, and 5-8 mice were included in each group. * $P < 0.05$; ** $P < 0.01$; *** $P < 0.001$; **** $P < 0.0001$ by 2-way ANOVA. μCT: micro-computed tomography; BV/TV: trabecular bone volume to total volume fraction; Tb.Th: trabecular thickness; Tb.N: Trabecular number; Tb.Sp: trabecular separation; Ct.BMD: cortical bone mineral density Ct.Th: cortical thickness; Ct.Ar: cortical area.

[1] Hunskaar S, Fasmer OB, Hole K. Formalin test in mice, a useful technique for evaluating mild analgesics. J Neurosci Methods 1985;14(1):69-76.

[2] Kanui TI, Tjolsen A, Lund A, Mjellem-Joly N, Hole K. Antinociceptive effects of intrathecal administration of alpha-adrenoceptor antagonists and clonidine in the formalin test in the mouse. Neuropharmacology 1993;32(4):367-371.
